# Clustering by multiple long-term conditions and social care needs: a cross-sectional study among 10 026 older adults in England

**DOI:** 10.1136/jech-2023-220696

**Published:** 2023-08-23

**Authors:** Nusrat Khan, Christos V Chalitsios, Yvonne Nartey, Glenn Simpson, Francesco Zaccardi, Miriam Santer, Paul J Roderick, Beth Stuart, Andrew J Farmer, Hajira Dambha-Miller

**Affiliations:** 1 Primary Care Research Centre, University of Southampton, Southampton, UK; 2 Leicester Real World Evidence Unit, Leicester Diabetes Centre, University of Leicester, Leicester, UK; 3 Centre for Evaluation and Methods, Wolfson Institute of Population Health, Queen Mary University of London, London, UK; 4 Nuffield Department of Primary Care Health Sciences, University of Oxford, Oxford, UK

**Keywords:** EPIDEMIOLOGY, GERIATRICS, PUBLIC HEALTH, CLUSTER ANALYSIS

## Abstract

**Background:**

People with multiple long-term conditions (MLTC) face health and social care challenges. This study aimed to classify people by MLTC and social care needs (SCN) into distinct clusters and quantify the association between derived clusters and care outcomes.

**Methods:**

A cross-sectional study was conducted using the English Longitudinal Study of Ageing, including people with up to 10 MLTC. Self-reported SCN was assessed through 13 measures of difficulty with activities of daily living, 10 measures of mobility difficulties and whether health status was limiting earning capability. Latent class analysis was performed to identify clusters. Multivariable logistic regression quantified associations between derived MLTC/SCN clusters, all-cause mortality and nursing home admission.

**Results:**

Our study included 9171 people at baseline with a mean age of 66.3 years; 44.5% were men. Nearly 70.8% had two or more MLTC, the most frequent being hypertension, arthritis and cardiovascular disease. We identified five distinct clusters classified as high SCN/MLTC through to low SCN/MLTC clusters. The high SCN/MLTC included mainly women aged 70–79 years who were white and educated to the upper secondary level. This cluster was significantly associated with higher nursing home admission (OR=8.71; 95% CI: 4.22 to 18). We found no association between clusters and all-cause mortality.

**Conclusions:**

We have highlighted those at risk of worse care outcomes, including nursing home admission. Distinct clusters of individuals with shared sociodemographic characteristics can help identify at-risk individuals with MLTC and SCN at primary care level.

**What is already known on this topic**

- While it is established that multiple long-term conditions (MLTC) are linked to an increased risk of hospitalisation, nursing home admission and mortality, no previous research has examined this risk in relation to clusters of MLTC and social care needs (SCN) in England.

**What this study adds**

- Using latent class analysis, this study identified five clusters by MLTC and SCN with distinct characteristics and quantified their relationship with nursing home admission and mortality.

**How this study might affect research, practice or policy**

- The findings permit the identification of high-risk groups who are more likely to have worse care outcomes, including nursing home admission in the future. This can inform targeted preventive action to where it is most needed among those with MLTC. Recognition of MLTC and SCN clusters may also aid clinicians in moving away from a single disease management approach in older adults.

## Introduction 

The growing burden of multiple long-term conditions (MLTC) is a significant global challenge for health and social care systems.^1^ MLTC is defined as the co-existence of two or more long-term conditions. One in four people worldwide is estimated to have a MLTC, although prevalence rises with age, from 54% in those over 65 years of age to 83% in those over 85 years.^2 3^

People living with MLTC require more intensive and complex person-centred care over a longer period than those with a single condition, which increases service utilisation and care costs due to the holistic nature of multiple diseases for specialised treatment requirements.^4^ Earlier studies have shown that those with MLTC aged between 50 and 64 years report difficulty with activities of daily living (ADL) and mobility in 15% and 18%, respectively.^5^ A recent analysis of the Health and Retirement Study in China found that nearly one-quarter of participants with MLTC developed difficulty with one or more ADLs during middle age.^6^ MLTC also increases the likelihood of frailty, reduced mobility and a general functional decline that often significantly impairs personal independence. In turn, this has increased the demand for social care, including higher levels of admissions to nursing or care homes, increased need for assisted living and a growth in ‘homecare’ support services to enable people to live independently as long as possible.^7 8^ Earlier studies have linked MLTC to a higher risk of hospitalisation, nursing home admission and mortality.^3 9^

Given the growing numbers of people presenting with complex social care needs (SCN) and the increased burden of MLTC, clustering approaches could present a strategy for identifying those with specific combinations of MLTC and SCN who are at risk of adverse health outcomes. Over 50% of people with MLTC will also have SCN so considering them in combination could be helpful in delivering holistic targeted interventions.^10^ Clustering relies on the fact that common conditions group together in predictable patterns within a population.^11 12^ Latent class analysis (LCA) has been a commonly used algorithm to identify clusters among people with MLTC.^13 14^ Clustering by both MLTC and SCN may allow more precise identification of those who could benefit most from preventive interventions and increased resource allocation in a holistic way.^15^ Application of advanced model-based clustering approaches, such as LCA, can derive unique clusters with specific combinations of MLTC and SCN and shared sociodemographic characteristics identifying at-risk at the primary care level.^12^

Although some advances have been made in MLTC clustering research,^16 17^ there is a scarcity of evidence considering SCN in combination with MLTC. This study aimed to classify people by MLTC and SCN into distinct clusters and quantify the association between derived clusters and care outcomes.

## Methods 

### Data source  

The English Longitudinal Study of Ageing (ELSA) is a study of people aged 50 years or older living in England.^18^ Briefly, a population-representative sample of members was drawn from the Health Survey for England (HSE) from 2002, with repeated waves of follow-up every 2 years and additional nurse visits to assess biomarkers every 4 years.^18 19^ It included 12 099 people in 2002 as the study entry point, with a wide range of data collected on physical and mental health, well-being, finances and attitudes around ageing over time. ELSA is an open cohort, and refreshment samples have been added by corresponding HSE surveys depending on the proportional age requirement for ELSA (eg, 50–74 years and their partners for wave 4 and 50–53 years and partners for wave 9), using cross-sectional and longitudinal weights for the core surveyed. The datasets of ELSA harmonised (elsa_harmonised) and ELSA harmonised G2 (elsa_harmonised_g2) were used for this study.

### Study design and population  

This cross-sectional study uses ELSA wave 2 (2002/2003) to wave 9 (2018/2019), with or without MLTC. Our baseline was wave 2, which included data from nurse visits, allowing more MLTC to be included and verified by nurse records rather than relying on self-reported data. This study was conducted, and findings were reported in line with the Strengthening the Reporting of Observational Studies in Epidemiology guidelines for observational studies using routinely collected health data.^19^

### Multiple long-term conditions

Ten MLTC were identified in the ELSA datasets based on our previous works and consensus on defining MLTC, which identified a total of 59 long-term conditions (online supplemental table 1).^15 20 21^ The 10 conditions available in ELSA included hypertension, diabetes, cancer, lung disease, cardiovascular disease, stroke, mental health disorders, arthritis, Parkinson’s disease and dementia. The presence of these conditions is defined in ELSA by self-reporting and nurse review of healthcare records.^18^ Due to the small sample size (less than 10 cases), some conditions were combined following clinical discussion and consensus: depression was included among mental health disorders, asthma within lung disease, Alzheimer’s disease within dementia, heart attack, angina, heart murmur, abnormal heart rhythm and congestive heart failure within cardiovascular diseases. We considered the highest number of MLTC developed by each participant across multiple waves.

10.1136/jech-2023-220696.supp1 Supplementary data

### Social care needs  

SCN variables were identified by a parallel Delphi consensus study that included professionals, people living with MLTC and informal carers identifying SCN in MLTC.^5 20^ Variables identified from the Delphi were mapped to the ELSA data dictionary, resulting in an operational definition of SCN as follows: (1) 13 self-report (yes/no) difficulties in ADL; (2) 10 self-report binary (yes/no) difficulty in physical mobility and (3) self-report on whether an individual’s health status was limiting earning capability.^22^ The ELSA questionnaire included standardised measures for quantifying ADL and mobility variables, which have undergone extensive validation in previous studies.^23^ The ADL variables included: difficulty with dressing; putting on shoes and socks; walking across a room; bathing or showering; eating such as cutting up food; getting in and out of bed; using the toilet; getting up or down; using a map for location; preparing a hot meal; shopping for groceries; making telephone calls; taking medications; doing work around house and garden; managing money, eg, paying bills and keeping track of expenses. The mobility variables included: difficulty in the ability to walk 100 yards; sit for 2 hours; get up from the chair after sitting for prolonged periods; climb several flights of stairs without resting; climb one flight of stairs without resting; kneel or crouch; reach or extend arms above shoulder level; pull or push large objects; lift or carry weights over 10 pounds; picking up 5 p coin from a table. Health status limiting earning capability was a variable that was also included under our definition of SCN. It denotes whether an impairment or health problem limits the type or amount of paid employment.^18 22^ We combined the 13 items of ADL and the 10 items of difficulty with physical mobility into one composite score for ADL and mobility. For each item, a score was assigned for the absence and presence of ADL and mobility difficulties, respectively (score 0 if absence and score 1 if presence). Therefore, the overall sum of scores across all items was either 0 or ≥1. Those with a sum of ≥1 was considered to have ADL or mobility difficulties. For our SCN variable, we considered the maximum number of SCN developed by each participant during the study period.   

### Care outcomes 

The outcomes of interest were nursing home admission and all-cause mortality of the participants. These were self-reported with end-of-life or after-death interviews on waves 2, 3, 4 and 6 among a sample of family members or carers of ELSA participants who had recently passed away, asking about the circumstances around the respondent’s final stages of life.^18 19^

### Sociodemographic

Self-reported information was available at baseline for age (continuous), sex and ethnicity (grouped within the database as whites or non-whites). Age was further categorised for analysis (50–59, 60–69, 70–79, ≥80 years old). Education level was categorised into four groups: less than upper secondary level, upper secondary and vocational level, tertiary level and others. Employment status was categorised as working for payment and not working for payment. Marital status was categorised into three groups: never married, married/having a partner and separated/divorced/widowed. To minimise the impact of missing data, we used data provided in the subsequent waves for any missing information at baseline. 

### Statistical analysis 

We summarised the characteristics of those with and without MLTC using descriptive statistics. LCA was conducted to identify distinct clusters of MLTC and SCN. LCA is a model-based clustering technique that classifies individuals into clusters based on multiple characteristics (in this case, MLTC and SCN).^24^ The posterior probability of belonging to each cluster can be obtained for each participant; assigned according to their highest probability of membership. The underlying assumption of LCA is that individuals belong to unobserved (latent) clusters but can be classified based on information available in observed data through a likelihood function. A series of latent class models were fitted iteratively, beginning with two clusters and up to six clusters. Six clusters were the maximum fitted to balance optimal fit with clinical utility. The optimal number of latent clusters was determined using the dissimilarity index and the Bayesian Information Criterion (BIC) as robust indicators of the cluster alongside clinical interpretation.^25 26^ BIC was used to compare several plausible models with the lowest values to indicate the best-fitting model. Kruskal-Wallis and χ^2^ tests were employed to compare the characteristics of the clusters. Multivariable logistic regression was computed to assess the association of each MLTC/SCN cluster with the outcomes (nursing home admission and all-cause mortality), adjusted for age, sex, ethnicity, marital status, education and employment. The cluster with the highest number of people was considered the reference category. As a sensitivity analysis, we also considered the ‘healthiest’ cluster as the reference category. Data management and analyses were conducted using Stata M.P. (V.17).

## Results 

### Characteristics of the study population

A total of 9171 people were identified at baseline (wave 2). They were mainly white (98%) and women (55.5%), with a mean (SD) age of 66.3 (10) years (table 1). Among them, individuals with MLTC comprised 70.8% (table 1). Most were married or partnered (66.4%), 11.2% completed level 3 upper education and nearly two-thirds (72.9%) were not working. At baseline, 36.8% of those with MLTC had at least one ADL difficulty, and 68.6% had at least one mobility difficulty (table 1). A total of 499 individuals died during the follow-up period (all-cause mortality 3.9%), with 24.9% (n=134) having stayed in a nursing home.

**Table 1:** Characteristics at baseline (wave 2)

	Total (n=9171)	MLTC (6489, 70.8%)	No MLTC (2682, 29.2%)
Age, years, mean (SD)	66.3 (10)	67.5 (9.8)	63.4 (9.7)
Age, years			
50–59	2925 (31.9)	1739 (26.8)	1186 (44.2)
60–69	2920 (31.8)	2091 (32.2)	829 (30.9)
70–79	2203 (24)	1775 (27.3)	428 (16)
≥80	1123 (12.2)	884 (13.6)	239 (8.9)
Sex			
Male	4084 (44.5)	2791 (43)	1293 (48.2)
Female	5087 (55.5)	3698 (57)	1389 (51.8)
Ethnicity			
White	8963 (98)	6341 (97.7)	2622 (97.8)
Non-white	206 (2)	148 (2.3)	58 (2.2)
Missing*	2 (0.02)	0 (0)	2 (0.1)
Marital status			
Married/partnered	6335 (69.1)	4308 (66.4)	2027 (75.6)
Separated/divorced/widowed	2411 (26.3)	1890 (29.1)	521 (19.4)
Never married	424 (4.6)	291 (4.5)	134 (5)
Missing*	1 (0.01)	0 (0)	1 (0.04)
Education†			
Less than upper secondary	3563 (38.8)	2681 (41.3)	882 (32.9)
Upper secondary and vocational training	3687 (40.2)	2483 (38.3)	1204 (44.9)
Tertiary education	1124 (12.3)	724 (11.2)	400 (14.9)
Other	784 (8.5)	596 (9.2)	188 (7)
Missing*	13 (0.1)	5 (0.1)	8 (0.3)
Employment			
Currently working	3075 (33.5)	1755 (27.1)	1320 (49.2)
Not working	6095 (66.5)	4734 (72.9)	1361 (50.7)
Missing*	1 (0.01)	0 (0)	1 (0.04)
Long-term conditions			
Diabetes	744 (8.1)	718 (11.1)	26 (1.0)
Hypertension	3793 (41.3)	3396 (52.3)	397 (14.8)
Cancer	662 (7.2)	607 (9.4)	62 (2.3)
Lung diseases	1503 (16.4)	1387 (21.4)	116 (4.3)
Cardiovascular diseases	1981 (21.6)	1851 (28.5)	130 (4.9)
Stroke	442 (4.8)	429 (6.6)	13 (0.5)
Mental health disorders	1964 (21.4)	1818 (28.0)	146 (5.5)
Arthritis	3207 (35)	2919 (45.0)	288 (10.7)
Parkinson disease	54 (0.6)	50 (0.8)	4 (0.2)
Dementia	77 (0.7)	70 (1.1)	7 (0.3)
Social care needs			
Difficulties in any ADL	2716 (29.6)	2390 (36.8)	326 (12.2)
Difficulty in any physical mobility	5423 (59.1)	4454 (68.6)	969 (36.2)
Health status limiting earning capability	2990 (32.6)	2620 (40.4)	370 (14.0)

All figures are expressed as absolute numbers and percentages unless otherwise specified.

*Missing=missing+not available+no respondent.

†Upper secondary=level 3 secondary education. Typically aged 16–18 years.

ADL, activities of daily living; MLTC, multiple long-term conditions.

### Clustering MLTC and SCN 

**Figure 1:**
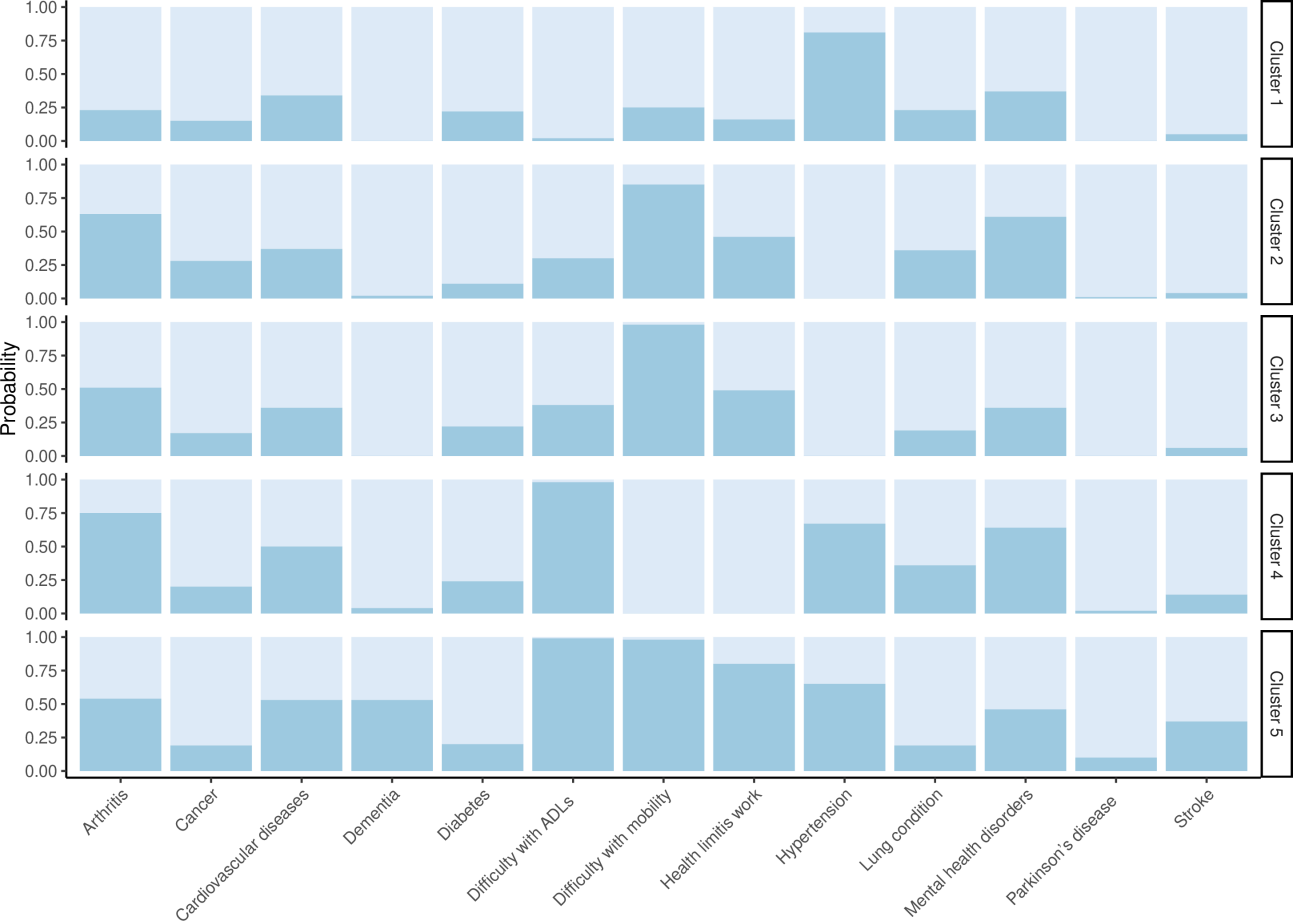
Item response probabilities of multiple long-term conditions and social care needs clusters.

We applied LCA in a total of 10 026 people with MLTC who had complete data from wave 2 to wave 9 (online supplemental figure 1). Based on the lowest BIC (online supplemental table 2), five distinct clusters were identified (figure 1 & online supplemental table 3). The dissimilarity index was 0.25. Cluster 1 (9.3%, n=934) represented the highest probability of hypertension (81%), cardiovascular disease (34%) and mental health disorder (37%). In cluster 2 (13.7%, n=1370), 85% of people had a high probability of mobility difficulty, followed by arthritis, mental health disorders and cardiovascular diseases. Cluster 3 (21.9%, n=2197) was dominated by a high probability of SCN conditions, with 98% of mobility difficulties and 49% with health status limiting work. Cluster 4 (49.2%, n=4937) was also dominated by ADL difficulties with a probability of 98%, followed by 75% of arthritis and 67% of hypertension. However, cluster 5 (5.9%, n=587) was prominently dominated by all the SCN, with a 99% probability of ADL difficulties, 98% of mobility difficulties and 80% of health status limiting earning capability. All the clusters were dominated by arthritis, mental health disorders, cardiovascular diseases and hypertension in terms of disease conditions.

In terms of sociodemographic characteristics of the clusters, cluster 1 had the youngest median (IQR) age of 57 (53–65) years. Individuals in cluster 5 (dominated by all three SCN variables) had the highest median age (IQR) of 75 years (69–81) (table 2). In terms of ethnicity, all the clusters had a majority of white individuals. Cluster 1 had the highest proportion of non-white individuals (6%), and cluster 5 had the lowest (2.4%). All the clusters had more women than men except for cluster 1. The clusters differed by marital status and education; specifically, from low SCN/MLTC cluster 1 to high SCN/MLTC cluster 5, the proportion of separated/divorced/widowed people increased from 13.5% to 32.5%. A trend of a lower educational level was observed with progression from low SCN to high SCN. Only 23% of individuals in the low SCN/MLTC cluster 1 had less than upper secondary level education compared with nearly 50% in the high MLTC/SCN cluster 5. Another low SCN/MLTC cluster (cluster 4) had a very high proportion of individuals who were previously married (31%) and unemployed (79%). About 21.5% of the individuals received tertiary education in cluster 1 and 18.5% in cluster 2 compared with a much lower 8.7% in cluster 5.

**Table 2:** Sociodemographic characteristics by multiple long-term conditions and social care needs clusters

Characteristics	Cluster 1 (n=934; 9.3%)	Cluster 2 (n=1371; 13.7%)	Cluster 3 (n=2197; 21.9%)	Cluster 4 (n=4937; 49.2%)	Cluster 5 (n=587; 5.9%)
Age (years), median (IQR)	57 (53–65)	59 (54–67)	61 (56–69)	66 (58–74)	75 (69–82)
Age (years)					
50–59	182 (41.2)	288 (37.7)	479 (34.4)	763 (22.2)	27 (5.9)
60–69	166 (37.6)	285 (37.4)	486 (34.9)	1065 (31.0)	89 (19.3)
70–79	83 (18.8)	143 (18.7)	312 (22.4)	1056 (30.8)	181 (39.3)
80+	11 (2.5)	47 (6.2)	117 (8.4)	546 (15.9)	163 (35.4)
Sex					
Male	596 (63.7)	570 (41.6)	1021 (46.5)	2086 (42.3)	262 (44.6)
Female	339 (36.3)	800 (58.4)	1176 (53.5)	2851 (57.7)	325 (55.4)
Ethnicity					
White	879 (94.0)	1320 (96.4)	2104 (95.8)	4759 (96.4)	573 (97.6)
Non-white	56 (6.0)	50 (3.6)	93 (4.2)	177 (3.6)	14 (2.4)
Marital status					
Married/partnered	763 (81.6)	1034 (75.5)	1638 (74.6)	3106 (62.9)	374 (63.7)
Separated/divorced/widowed	126 (13.5)	262 (19.1)	449 (20.4)	1537 (31.1)	194 (33.0)
Never married	46 (4.9)	73 (5.3)	110 (5.0)	294 (6.0)	19 (3.2)
Education					
Less than upper secondary	213 (23.3)	345 (25.4)	687 (31.6)	2221 (45.4)	284 (48.8)
Upper secondary and vocational training	453 (49.4)	657 (48.3)	961 (44.2)	1842 (37.6)	195 (33.5)
Tertiary education	197 (21.5)	252 (18.5)	352 (16.2)	407 (8.3)	54 (9.3)
Other	54 (5.9)	106 (7.8)	172 (7.9)	427 (8.7)	49 (8.4)
Employment					
Not working	354 (37.9)	612 (44.7)	1159 (52.8)	3891 (78.8)	534 (91.0)
Currently working	580 (62.1)	757 (55.3)	1038 (47.2)	1045 (21.2)	53 (9.0)

The numbers are presented as absolute numbers and percentages unless otherwise specified.

The clusters differ, statistically significant (p<0.0001).

### MLTC/SCN clusters and care outcomes

**Figure 2:**
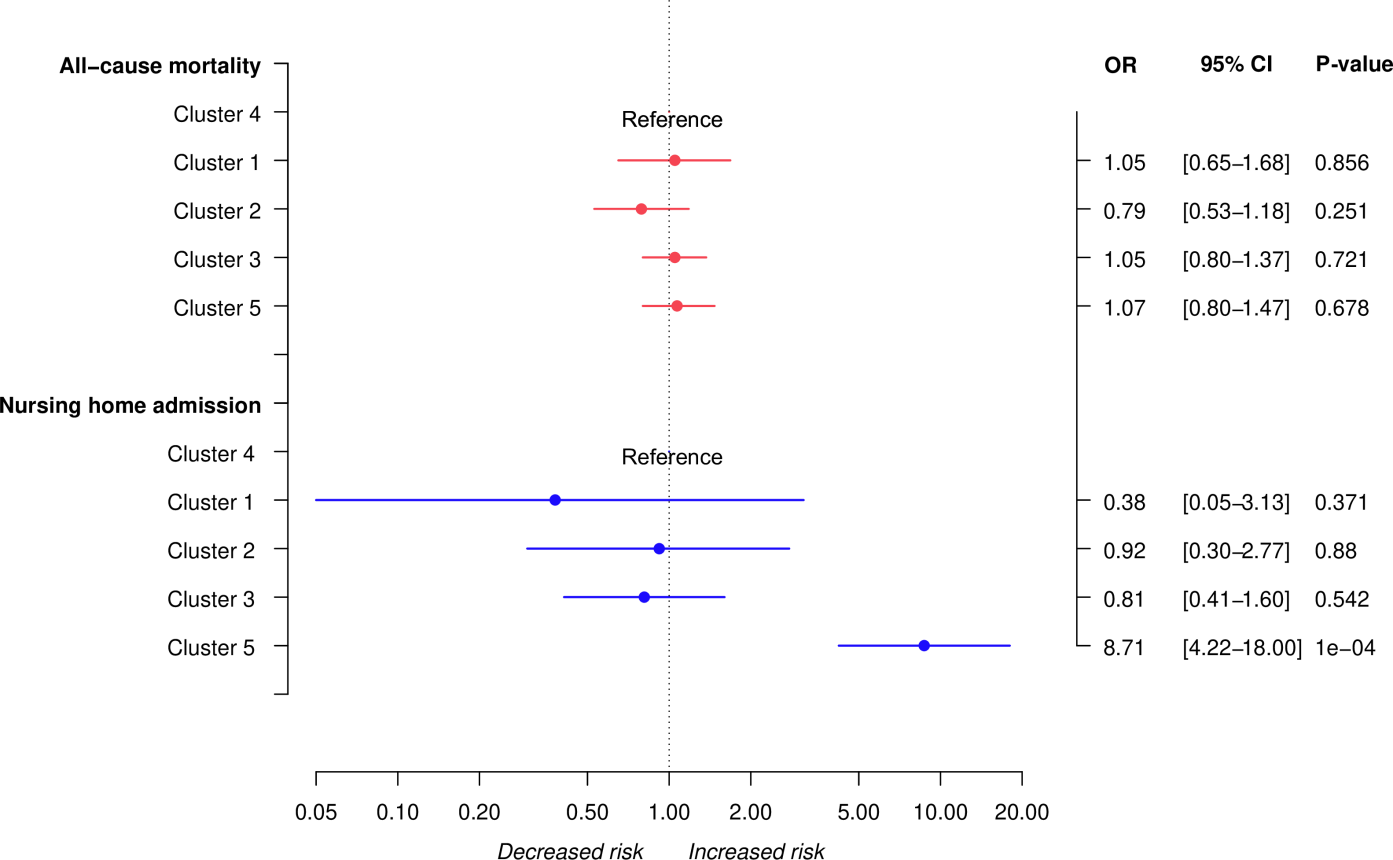
ORs (with 95% CI) of the association between clusters of multiple long-term conditions/social care needs and the outcomes of interest.

Cluster 5 had higher odds of nursing home admission (aOR=8.97; 95% CI: 4.36 to 18.45) compared with cluster 4, the cluster with the highest number of people, and none of the clusters was associated with all-cause mortality (figure 2, online supplemental tables 4 and 5). We found identical results when we considered the ‘healthiest’ cluster as the reference category (online supplemental tables 6 and 7).

## Discussion   

We established five distinct MLTC/SCN clusters among 10 025 older adults in England. Among the 10 conditions that were examined, only a combination of arthritis, mental health disorders, cardiovascular diseases and hypertension dominated when combined with SCN. This specific combination of MLTC and high SCN (cluster 5) was associated with a higher risk of nursing home admission than the cluster 4, the cluster with the highest number of people, after adjusting for confounders, including age. People with a lower level of education, unemployed or separated/divorced/widowed were more likely to fall into cluster 4.

One of the major current and upcoming difficulties for healthcare systems globally has been identified as MLTC.^27^ To tailor healthcare design, broad general descriptions of the health outcomes and demands of patients with MLTC (ie, based on counts of conditions) are not helpful. As a result, there have recently been calls to shift away from merely counting diseases in favour of a more specialised comprehension of which medical problems are most likely to co-occur (eg, clustering of diseases).^28^ Therefore, to deliver optimal care to possible homogenous patient population groups, there has been increasing interest in focusing research on clinically meaningful clusters and wider determinants instead of considering MLTC as a general concept.^27 29 30^ In clinical practice, the increased strain on services, staff shortages and rapid growth in the number of people with MLTC also mean that preventive measures and interventions at a population level are not applied routinely in practice and are costly. By considering specific clusters, clinicians may be more likely and able to deliver targeted care where is most needed—in a more efficient and cost-effective way.

Clustering by social care or wider health determinants remains limited in the present literature.^31^ Most observational research to date in MLTC has focused on the co-occurrence of conditions and biological determinants.^25 26 32^ Two previous systematic reviews summarise the literature on MLTC clusters and highlight that this is primarily described by the co-occurrence of conditions, including cardiovascular diseases and mental health disorders.^32 33^ We also observed this in our findings, although this was additionally accompanied by arthritis and hypertension. A recent longitudinal study of 16 years among older adults in Taiwan revealed that the cardiometabolic MLTC pattern had a much stronger association with increased mortality.^34^ A recent comparative study on MLTC clusters in the USA, Canada, England and Ireland showed the patterns of disease clusters and the risk factors related to each disease cluster were similar; however, the probabilities of the diseases within each cluster differed across countries.^34^ This highlights the necessity of identifying different clusters of MLTC and conditions with high probabilities to co-occurrence.^34 35^ MLTC, in general, has been explored widely to identify sociodemographic risk factors. Distinct sociodemographic characteristics of MLTC clusters have also been identified in a limited number of studies. In a large-scale 16 year longitudinal study in Brazil, women and men presented different mortality patterns according to MLTC combinations.^36^ Four longitudinal studies from electronic health records in the USA, UK, Europe and China highlighted the role of marital relationships in shaping the trajectory of health and well-being across the life course in people with MLTC.^37^ However, the generalisation of MLTC research findings in various contexts is complex, given the multimodal nature. Rather than managing all patients with MLTC and SCN the same, our findings show that it is possible to identify at-risk individuals according to their individual MLTC and SCN. This will highlight to general practitioners those who are at risk of worse care clinical outcomes and nursing home admission. In turn, more targeted prevention and increased follow-up could be initiated to reduce some of the outcomes.

To our knowledge, this is the first study that has examined clustering by both MLTC and SCN in England. The major strengths of our study are the use of the ELSA data, which are nationally representative of people aged 50 years and older and the implication of multiple measures of SCN for a more reliable understanding of this concept. Some limitations of our study should also be acknowledged. First, we used cross-sectional data; therefore, causality cannot be inferred. Second, many of the variables were derived through self-report health and social care assessment, which may be subject to information and recall bias. The analysis used only 10 MLTC based on what was available in the ELSA data, so a different association might have arisen if other MLTC or SCN had been considered. Additionally, when interpreting the results of observational studies, the sample might only represent healthy survivors in the population. Finally, the reverse causality between MLTC and ADL and mobility difficulties could not be addressed, although there is an abundance of previous literature reporting on this direction of association.^7 23 38^

Current literature calls^39^ for more work on holistic clusters considering wider determinants to deliver optimal care. Application of advanced model-based clustering approach (eg, LCA) can derive unique clusters with specific combinations of MLTC and SCN and highlight those who are at risk of worse care outcomes, including nursing home admission.^26 40^ This has important policy and clinical practice implications as it may allow more precise identification of those who could benefit most from preventive measures.^16 35 36^ Previous studies have shown that older people with MLTC and social needs are likely to have worse health outcomes,^31 34^ but our data provide more specific combinations of conditions which were statistically and sociodemographically distinct. Identifying target populations with complex MLTC clusters can further build better health and social care system models and interventions that better integrate the clinical management of MLTC while concurrently addressing SCN.

In conclusion, we identified SCN/MLTC clusters with varying health and social demand and were able to differentiate between clusters by sociodemographic characteristics. We also showed that care outcomes could vary by cluster. Further research will need to explore the temporality of these associations and examine long-term outcomes beyond nursing home admission and mortality, including economic analysis.

## Data Availability

Data may be obtained from a third party and are not publicly available. ELSA data were available through the UK Data Archive and are widely available to access in this way; as such, our study data will not be made available for access.
