# Correction: Clustering by multiple long-term conditions and social care needs: a cross-sectional study among 10 026 older adults in England

**DOI:** 10.1136/jech-2023-220696corr1

**Published:** 2024-08-09

Khan N, Chalitsios CV, Nartey Y, *et al.* Clustering by multiple long-term conditions and social care needs: a cross-sectional study among 10 026 older adults in England. *J Epidemiol Community Health* 2023;**77**:770–776. Additional funding information is required for one of the authors, Andrew Farmer. The correct funding statement is:

The research was funded/supported by the National Institute for Health Research (NIHR) Oxford Biomedical Research Centre (BRC). The views expressed are those of the author(s) and not necessarily those of the NHS, the NIHR or the Department of Health.

