# Supplementary material for: Clustering by multiple long-term conditions and social care needs: a cross-sectional study among 10 026 older adults in England
Source: J Epidemiol Community Health. 2023 Aug 23;77(12):770–6. doi: 10.1136/jech-2023-220696 (PMC10646893; doi:10.1136/jech-2023-220696)
Supplement: Supplementary data [file jech-2023-220696supp001.pdf]

Clustering by Multiple Long-Term Conditions and Social Care Needs: A cohort study amongst 10,026 older adults in England

Nusrat Khan[1], Christos V. Chalitsios[1], Yvonne Nartey[1],Glenn Simpson[1], Francesco Zaccardi[2], Miriam Santer[1], Paul Roderick[1], Beth Stuart[4], Andrew Farmer[4], Hajira Dambha-Miller[1]

Supplementary

Supplementary Table 1. List of 59 long-term conditions based on our previous works and consensus on defining multiple long-term conditions.

**Long-term conditions**
Stroke
Coronary heart disease
Heart failure
Peripheral arterial disease
Heart valve disorder
Arrhythmia
Venous thromboembolism
Aneurysm
Hypertension (Treated and untreated)
Diabetes
Addison's disease
Cystic fibrosis
Thyroid disorders
COPD
Asthma
Bronchiectasis
Parkinson's disease
Epilepsy
Multiple sclerosis
Paralysis
Transient ischaemic attack
Peripheral neuropathy
Chronic primary pain
Solid organ cancer
Haematological cancer
Metastatic cancer
Melanoma
Benign cerebral tumours causing disability
Dementia
Schizophrenia
Depression
Anxiety
Bipolar disorder
Drug or alcohol misuse
Eating disorder
Autism

Post-traumatic stress disorder
Connective tissue disorder
Osteoarthritis
Osteoporosis
Gout
Long-term musculoskeletal problem due to injury
Chronic liver disease
Inflammatory bowel
Chronic pancreatic disease
Peptic ulcer
Chronic kidney disease
End-stage kidney disease
Endometriosis
Chronic urinary tract infection
Anaemia (including pernicious anaemia and sickle cell anaemia)
Visual impairment that cannot be corrected
Hearing impairment that cannot be corrected
Meniere's disease
HIV/AIDS
Chronic lyme disease
Tuberculosis
Post-acute COVID-19 disease
Chromosomal abnormalities
Congenital heart disease

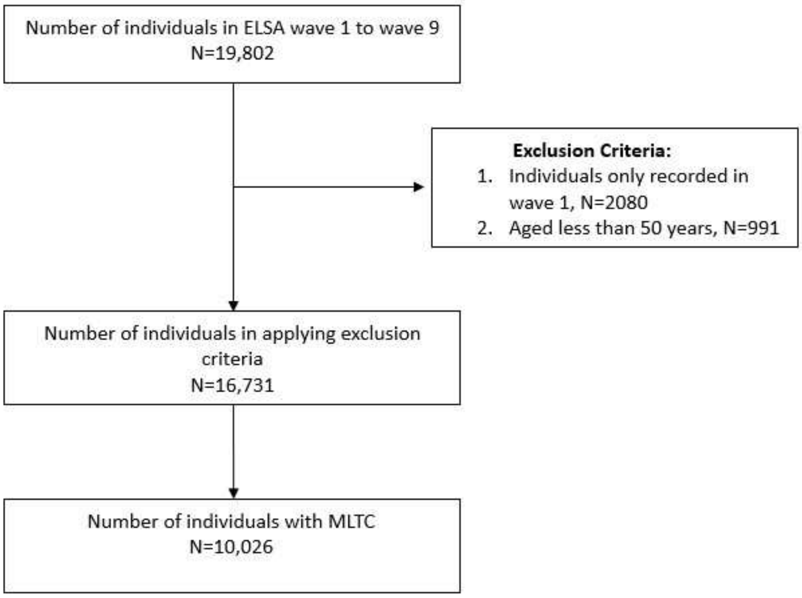

Supplementary Figure 1. Flow chart for the study population in ELSA.

Supplementary Table 2: Model fit and diagnostic criteria for competing latent class models.

	LL	BIC(LL)	AIC(LL)	Npar	df	Classification Error	Dissimilarity index	Entropy R^2
**1-Cluster**	-64952.8	130025.1	129931.7	13	8178	0	0.40	1
**2-Cluster**	-61986.9	124221.9	124027.8	27	8164	0.0717	0.28	0.7401
**3-Cluster**	-61733.4	123843.6	123548.8	41	8150	0.0736	0.27	0.7999
**4-Cluster**	-61575.3	123656	123260.5	55	8136	0.1224	0.26	0.7194
**5-Cluster**	-61421.7	123377.6	122781.5	69	8122	0.1688	0.25	0.6751
**6-Cluster**	-61293.3	123449.3	122952.5	83	8108	0.2082	0.24	0.6605

Abbreviations: AIC, Akaike Information Criterion; BIC, Bayesian Information Criterion; df, degrees of freedom; LL, Log Likelihood; Npar, Number of parameters

Supplementary Table 3: Item response probabilities (95%CI) of the clusters of multiple long-term conditions and social care needs.

Cluster 1		Cluster 2		Cluster 3		Cluster 4		Cluster 5	
**Condition/SCN**	**Probability**	**Condition/SCN**	**Probability**	**Condition/SCN**	**Probability**	**Condition/SCN**	**Probability**	**Condition/SCN**	**Probability**
Hypertension	0.81 (0.75 - 0.86)	Difficulty with mobility	0.85 (0.81 - 0.89)	Difficulty with mobility	0.98 (0.94 - 0.99)	Difficulty with ADLs	0.98 (0.94 - 0.99)	Difficulty with ADLs	0.99 (0.92 - 1.00)
Mental health disorders	0.37 (0.33 - 0.41)	Arthritis	0.63 (0.59 - 0.66)	Arthritis	0.51 (0.47 - 0.55)	Arthritis	0.75 (0.72 - 0.77)	Difficulty with mobility	0.98 (0.96 - 0.99)
Cardiovascular diseases	0.34 (0.31 - 0.38)	Mental health disorders	0.61 (0.58 - 0.64)	Health limit work	0.49 (0.44 - 0.55)	Hypertension	0.67 (0.65 - 0.69)	Health limit work	0.80 (0.74 - 0.85)
Difficulty with mobility	0.25 (0.14 - 0.42)	Health limit work	0.46 (0.41 - 0.51)	Difficulty with ADLs	0.38 (0.32 - 0.44)	Mental health disorders	0.64 (0.62 - 0.66)	Hypertension	0.65 (0.59 - 0.70)
Arthritis	0.23 (0.20 - 0.27)	Cardiovascular diseases	0.37 (0.34 - 0.40)	Mental health disorders	0.36 (0.34 - 0.39)	Cardiovascular diseases	0.50 (0.48 - 0.52)	Arthritis	0.54 (0.48 - 0.60)
Lung condition	0.23 (0.20 - 0.27)	Lung condition	0.36 (0.33 - 0.39)	Cardiovascular diseases	0.36 (0.33 - 0.38)	Lung condition	0.36 (0.34 - 0.38)	Cardiovascular diseases	0.53 (0.48 - 0.59)
Diabetes	0.22 (0.19 - 0.25)	Difficulty with ADLs	0.30 (0.25 - 0.35)	Diabetes	0.22 (0.19 - 0.24)	Diabetes	0.24 (0.22 - 0.26)	Dementia	0.53 (0.38 - 0.67)
Health limits work	0.16 (0.13 - 0.20)	Cancer	0.28 (0.25 - 0.31)	Lung condition	0.19 (0.16 - 0.21)	Cancer	0.20 (0.19 - 0.22)	Mental health disorders	0.46 (0.40 - 0.52)
Cancer	0.15 (0.13 - 0.18)	Diabetes	0.11 (0.09 - 0.14)	Cancer	0.17 (0.16 - 0.19)	Stroke	0.14 (0.12 - 0.16)	Stroke	0.37 (0.32 - 0.43)
Stroke	0.05 (0.04 - 0.07)	Stroke	0.04 (0.03 - 0.05)	Stroke	0.06 (0.04 - 0.07)	Dementia	0.04 (0.02 - 0.06)	Diabetes	0.23 (0.19 - 0.28)
Difficulty with ADLs	0.02 (0.01 - 0.05)	Dementia	0.02 (0.01 - 0.03)	Dementia	0.00 (0.00 - 0.02)	Parkinson's disease	0.02 (0.01 - 0.03)	Cancer	0.19 (0.15 - 0.24)
Parkinson's disease	0.00 (0.00 - 0.01)	Parkinson's disease	0.01 (0.00 - 0.02)	Parkinson's disease	0.00 (0.00 - 0.01)	Difficulty with mobility	0.00 (0.00 - 0.00)	Lung condition	0.19 (0.15 - 0.23)
Dementia	0.00 (0.00 - 0.00)	Hypertension	0.00 (0.00 - 0.00)	Hypertension	0.00 (0.00 - 1.00)	Health limit work	0.00 (0.00 - 0.00)	Parkinson disease	0.10 (0.07 - 0.13)

*ADL is a composite of 13 variables = difficulty dressing, including putting on shoes and socks, difficulty walking across a room, difficulty bathing or showering, difficulty eating, such as cutting up food, difficulty getting in and out of bed, difficulty using the toilet, including getting up or down, difficulty using map to figure out how to get around strange place, difficulty preparing a hot meal, difficulty shopping for groceries, difficulty making telephone calls, difficulty taking medications, difficulty doing work around house and garden, difficulty managing money, e.g. paying bills, keeping track expense

**Mobility is a composite of 10 variables = difficulty walking 100 yards, difficulty sitting for 2 hours, difficulty getting up from a chair after sitting for long periods, difficulty climbing several flights of stairs without resting, difficulty climbing one flight of stairs without resting, difficulty stooping, kneeling or crouching, difficulty reaching or extending arms above shoulder level, difficulty pulling or pushing large objects, difficulty lifting or carrying weights over 10 pounds, difficulty picking up 5p coin from the table

Supplementary Table 4. Association of SCN/MLTC clusters with all-cause mortality.

	Alive		Dead				
	N	%	N	%	Adjusted OR	95% CI	p-value
**Clusters**							
Cluster 4	4647	94.13	290	5.87	Reference		
Cluster 1	908	97.11	27	2.89	1.05	0.65-1.68	0.856
Cluster 2	1337	97.59	33	2.41	0.79	0.53-1.18	0.251
Cluster 3	2111	96.09	86	3.91	1.05	0.80-1.37	0.721
Cluster 5	524	89.27	63	10.7	1.07	0.80-1.47	0.678
**Age (years)**							
50-59	1700	97.76	39	2.24	Reference		
60-69	2027	96.94	64	3.06	0.91	0.59-1.40	0.673
70-79	1598	90.03	177	9.97	2.82	1.88-4.22	<0.0001
80+	704	79.64	180	20.3	6.78	4.44-10.40	<0.0001
**Sex**							
Male	4,263	94.00	272	5.99	Reference		
Female	5,264	95.87	227	4.13	0.52	0.42-0.64	<0.0001
**Ethnicity**							
White	9,143	94.89	492	5.11	Reference		
Non-white	383	98.21	7	1.79	0.81	0.37-1.78	0.601
**Marital status**							
Married/ Partnered	6,614	95.65	301	4.35	Reference		
Separated/ Divorced/ Widowed	2,386	92.91	182	7.08	0.88	0.70-1.11	0.284
Never married	526	97.05	16	2.95	0.61	0.35-1.08	0.088
**Education**							
Less than upper secondary	3,491	93.09	259	6.91	Reference		
Upper secondary and vocational training	3,959	96.37	149	3.62	0.80	0.63-1.01	0.062
Tertiary education	1,213	96.12	49	3.88	0.95	0.67-1.37	0.798
Other	767	94.93	41	5.07	0.86	0.60-1.23	0.406
**Employment**							
Not working	6,085	92.90	465	7.09	Reference		
Currently working	3,440	99.05	33	0.95	0.39	0.25-0.60	<0.0001

Adjusted for all sociodemographic variables when not stratified by these.

Supplementary Table 5. Association of SCN/MLTC clusters with nursing home admission.

	Alive		Dead				
	N	%	N	%	Adjusted OR	95% CI	p-value
**Clusters**							
Cluster 4	222	75.77	71	24.23	Reference		
Cluster 1	26	96.30	1	3.70	0.38	0.05-3.13	0.371
Cluster 2	28	84.85	5	15.15	0.92	0.30-2.77	0.88
Cluster 3	71	82.56	15	17.44	0.81	0.41-1.60	0.542
Cluster 5	21	33.33	42	66.67	8.71	4.22-18	<0.0001
**Age (years)**							
50-59	35	89.74	4	10.26	Reference		
60-69	60	92.31	5	7.69	0.75	0.17-3.34	0.701
70-79	150	83.80	29	16.20	1.50	0.45-5.02	0.511
80+	97	53.89	83	46.11	4.34	1.31-14.3	0.016
**Sex**							
Male	219	80.22	54	19.78	Reference		
Female	59.51	59.62	40.3	40.38	1.44	0.83-2.51	0.193
**Ethnicity**							
White	361	72.93	134	27.07	Reference		
Non-white	7	100.00	0	0.00	NA	NA	NA
**Marital status**							
Married/ Partnered	247	81.52	56	18.48	Reference		
Separated/ Divorced/ Widowed	111	60.99	71	39.01	2.36	1.32-4.22	0.004
Never married	10	58.82	7	41.18	3.52	0.92-13.5	0.067
**Education**							
Less than upper secondary	184	70.50	77	29.50	Reference		
Upper secondary and vocational training	114	76.00	36	24.00	1.01	0.57-1.79	0.967
Tertiary education	41	83.67	8	16.33	0.62	0.22-1.77	0.373
Other	28	68.29	13	31.71	0.90	0.36-2.26	0.829
**Employment**							
Not working	338	72.22	130	27.78	Reference		
Currently working	30	90.91	3	9.09	0.70	0.17-2.84	0.614

Adjusted for all sociodemographic variables when not stratified by these.

Supplementary Table 6. Association of SCN/MLTC clusters with all-cause mortality considering the "healthiest" cluster as the reference group.

	Alive		Dead				
	**N**	**%**	**N**	**%**	**Adjusted OR**	**95% CI**	**p-value**
**Clusters**							
Cluster 1	908	97.11	27	2.89	Reference		
Cluster 2	1,337	97.59	33	2.41	2.39	0.24-24.02	0.458
Cluster 3	2,111	96.09	86	3.91	2.10	0.24-18.19	0.499
Cluster 4	4,647	94.13	290	5.87	2.61	0.32-21.26	0.371
Cluster 5	524	89.27	63	10.7	22.7	2.59-198.4	0.005
**Age (years)**							
50-59	1,700	97.79	39	2.24	Reference		
60-69	2,027	96.94	64	3.06	0.74	0.17-3.34	0.701
70-79	1,598	90.03	177	9.97	1.50	0.45-5.02	0.511
80+	704	79.64	180	20.4	4.34	1.31-14.33	0.016
**Sex**							
Male	4,263	94	272	6	Reference		
Female	5,264	95.87	227	4.13	1.44	0.83-2.51	0.193
**Ethnicity**							
White	9,143	94.89	492	5.11	Reference		
Non-white	383	98.21	7	1.79	NA	NA	NA
**Marital status**							
Married/ Partnered	6,614	95.65	301	4.35	Reference		
Separated/ Divorced/ Widowed	2,386	92.91	182	7.09	2.36	1.32-4.22	0.004
Never married	526	97.05	16	2.95	3.51	0.92-13.5	0.067
**Education**							
Less than upper secondary	3,491	93.09	259	6.91	Reference		
Upper secondary and vocational training	3,959	96.37	149	3.63	1.01	0.57-1.79	0.967
Tertiary education	1,213	96.12	49	3.88	0.62	0.22-1.77	0.373
Other	767	94.93	41	5.07	0.90	0.36-2.26	0.829
**Employment**							
Not working	6,085	92.9	465	7.10	Reference		
Currently working	3,440	99.05	33	0.95	0.69	0.17-2.84	0.614

Adjusted for all sociodemographic variables when not stratified by these.

Supplementary Table 7. Association of SCN/MLTC clusters with nursing home admission considering the "healthiest" cluster as the reference group.

	Alive		Dead				
	**N**	**%**	**N**	**%**	**Adjusted OR**	**95% CI**	**p-value**
**Clusters**							
Cluster 1	26	96.3	1	3.7	Reference		
Cluster 2	28	84.9	5	15.1	0.76	0.42-1.36	0.354
Cluster 3	71	82.6	15	17.4	1.00	0.60-1.67	0..986
Cluster 4	222	75.8	71	24.2	0.96	0.59-1.54	0.856
Cluster 5	21	33.3	42	66.7	1.02	0.59-1.77	0.935
**Age (years)**							
50-59	35	89.7	4	10.3	Reference		
60-69	60	92.3	5	7.7	0.91	0.59-1.40	0.673
70-79	150	83.8	29	16.2	2.82	1.88-4.23	<0.0001
80+	97	53.9	83	46.1	6.78	4.44-10.4	<0.0001
**Sex**							
Male	219	80.22	54	19.8	Reference		
Female	149	65.07	80	34.9	0.52	0.42-0.64	<0.0001
**Ethnicity**							
White	361	72.93	134	27.1	Reference		
Non-white	7	100	0	0	0.81	0.67-1.78	0.601
**Marital status**							
Married/ Partnered	247	81.52	56	18.5	Reference		
Separated/ Divorced/ Widowed	111	60.99	71	39	0.88	0.70-1.11	0.284
Never married	10	58.82	7	41.2	0.61	0.35-1.08	0.088
**Education**							
Less than upper secondary	184	70.5	77	29.5	Reference		
Upper secondary and vocational training	114	76	36	24	0.80	0.63-1.01	0.062
Tertiary education	41	83.67	8	16.3	0.95	0.67-1.36	0.798
Other	28	68.29	13	31.7	0.86	0.60-1.23	0.406
**Employment**							
Not working	338	72.22	130	27.8	Reference		
Currently working	30	90.91	3	9.09	0.39	0.25-0.60	<0.0001

Adjusted for all sociodemographic variables when not stratified by these.
